# Theoretical proposal for an intergenerational educational program for the prevention of human papilloma virus (HPV): Design, methodology and evaluation

**DOI:** 10.1016/j.aprim.2025.103249

**Published:** 2025-03-08

**Authors:** Maria Lourdes Gonçalves-Fernández, Margarita Pino-Juste

**Affiliations:** a GIES-10 Research Group, Galicia Sur Research Institute (IIS Galicia Sur), SERGAS-UVIGO, Spain; b Department of Didactics, School Organization and Research Methods, Faculty of Education and Sport Sciences, University of Vigo, 36005 Pontevedra, Spain

*Dear Editor*,

Human papillomavirus (HPV) is one of the most prevalent sexually transmitted infections worldwide, responsible for approximately 70% of cervical cancer cases.^1^ Despite the availability of prophylactic vaccines and screening tests, barriers to access and knowledge persist, particularly in vulnerable communities. Health literacy is crucial for improving HPV prevention; however, many educational interventions are one-dimensional and do not take advantage of modern digital tools.^2^

The proposed design is based on the implementation of a structured program consisting of nine two-hour sessions, aimed at women of various ages in vulnerable community settings.^3^, ^4^ This program seeks to promote intergenerational dialogue as a key strategy for the transmission of knowledge and the reinforcement of preventive practices within communities. The specific structure and content of the program are summarized in Table 1.

**Table 1:** Summary of the educational program content.

Session	Topic	Objective	Key activity
1	Introduction to STIs	Identify common STIs and their risks.	Screening of an educational video.
2	Transmission mechanisms	Understand routes of STI transmission.	Creation of transmission maps in groups.
3	Reproductive anatomy	Recognize anatomical structures.	Creation of TikTok videos on anatomy.
4	Symptoms and early detection	Identify symptoms and encourage testing.	Role-playing scenarios for discussion.
5	Specifics of HPV	Deepen knowledge of HPV and prevention.	Designing educational brochures.
6	Prevention strategies	Promote preventive behaviors.	Gamified activity: “Virus Hunter.”
7	Diagnosis and stigma reduction	Address barriers to seeking medical care.	Discussion and creation of positive messages.
8	Treatment options	Inform about treatment and adherence.	Case study analysis in groups.
9	Consolidating knowledge	Reinforce sustainable health behaviors.	Reflective group session and video creation.

## Measurements and methodology

Digital tools such as TikTok, artificial intelligence, and gamification were integrated alongside group activities to foster participatory learning. The evaluation includes:

• *Pretest*: Initial measurement of knowledge about HPV.
• *Continuous evaluation*: Observation of participation, perception surveys, and analysis of materials created by participants.
• *Posttest*: Final assessment of the impact on knowledge, attitudes, and behaviors.

The use of digital platforms enhances accessibility to information and fosters intergenerational interaction, helping to reduce cultural and social barriers that hinder sexual health education.^5^

## Expected results

The implementation of the program is expected to generate:

1. Greater knowledge about HPV, its transmission modes, and prevention.
2. Increased acceptance of vaccination and screening tests.
3. Reduction of the stigma associated with sexually transmitted infections.
4. Sustainable use of digital tools for health education.
5. Strengthening of intergenerational support networks within communities.
6. Greater autonomy in making informed decisions about sexual health.

In the long term, the program has the potential to be replicated in other communities with similar characteristics, adapting to different levels of health literacy and access to technology.

## Discussion

Intergenerational education has proven to be an effective strategy for transmitting health knowledge and preventing diseases in various communities.^6^ By fostering interaction between generations, trust in shared information is strengthened, and cultural barriers that limit access to sexual health knowledge are reduced.

The use of digital tools such as TikTok and gamification reinforces active learning and encourages participation among young people, who tend to be more receptive to these formats.^5^ However, a key challenge is the digital divide, as not all communities have equal access to electronic devices and internet connectivity. To mitigate this issue, the program proposes combining digital strategies with in-person activities and printed resources tailored to different literacy levels.

Moreover, continuous program evaluation is essential to measure its impact and make necessary adjustments according to the needs of each community. The implementation of pilot studies is recommended to validate the effectiveness of the methodology and explore its applicability in different contexts.

## Conclusions

Intergenerational education combined with digital technologies represents an innovative strategy for improving sexual health literacy. Although it has not yet been implemented, previous studies indicate that this approach could facilitate HPV prevention and encourage participation in vaccination and screening programs. Conducting pilot studies will be essential to adapting the proposal to different contexts.

## Conflict of interest

The authors declare no conflicts of interest in relation to this study.
